# Temporal trends in misclassification patterns of measured and self-report based body mass index categories - findings from three population surveys in Ireland

**DOI:** 10.1186/1471-2458-10-560

**Published:** 2010-09-17

**Authors:** Frances Shiely, Ivan J Perry, Jennifer Lutomski, Janas Harrington, C Cecily Kelleher, Hannah McGee, Kevin Hayes

**Affiliations:** 1 Department of Epidemiology and Public Health, University College Cork, Brookfield Health Sciences Complex, College Road, Cork, Ireland; 2 Department of Global Health, University of Washington, Harborview Medical Center, Seattle, USA; 3 School of Public Health, Physiotherapy and Population Science, University College Dublin, Woodview House, Belfield, Dublin 4, Ireland; 4 Population Health Sciences (Psychology), Royal College of Surgeons in Ireland, 123 St. Stephen's Green, Dublin 2, Ireland; 5 Department of Mathematics and Statistics, University of Limerick, Limerick, Ireland

## Abstract

**Background:**

As the use of self-reported data to classify obesity continues, the temporal change in the accuracy of self-report measurement when compared to clinical measurement remains unclear. The objective of this study was to examine temporal trends in misclassification patterns, as well as sensitivity and specificity, of clinically measured versus self-report based body mass index (BMI) from three national lifestyle surveys over a 10-year period.

**Methods:**

The Surveys of Lifestyle Attitudes and Nutrition (SLÁN) were interview based cross-sectional survey/measurements involving nationally representative samples in 1998, 2002 and 2007. Data from a subsample of both self-reported and measured height and weight were available from 66 men and 142 women in 1998, 147 men and 184 women in 2002 and 909 men and 1128 women in 2007. Respondents were classified into the BMI categories normal (< 25 kg m^-2^), overweight (25- < 30 kg m^-2^) and obese (≥ 30 kg m^-2^).

**Results:**

Underreporting of BMI increased across the three surveys (14%→21%→24%; p = 0.002). Sensitivity scores for the normal category exceeded 94% in all three surveys but decreased for the overweight (75%→68%→66%) and obese categories (80%→64%→53%). Simultaneously, specificity levels remained high.

**Conclusions:**

BMI values based on self-reported determinations of height and weight in population samples are underestimating the true prevalence of the obesity epidemic and this underestimation is increasing with time. The decreased sensitivity and consistently high specificity scores in the obese category across time, highlights the limitation of self-report based BMI classifications and the need for simple, readily comprehensible indicators of obesity.

## Background

Accurate measurements of height and weight typically cannot be made on all subjects participating in large epidemiological studies. Instead self-reported values of these variables must suffice, which although time and cost-efficient, are less precise and have no guarantee of accuracy[1]. Problems of precision and accuracy are further compounded when the variables height and weight are converted into biomedical measures such as body mass index (BMI = weight (kg)/[height (m)]^2^) [2].

Various reporting error patterns associated with these variables have been identified. The prevalence of overweight and obesity are generally underestimated when calculated from self-reported data compared to measured data [3-9]. Adults have been shown to systematically overestimate their height, irrespective of gender[3,4,10,11] and in general, weight is underestimated for both women and men [3,10-12]. The extent of underreporting of weight increases with increasing measured weight [3,13,14]. Many studies focus their comparative analyses of self-report and measured BMI on obese subjects (BMI ≥ 30 kg m^-2^) only [4], or overweight (25 ≥ BMI < 30 kg m^-2^) and obese subjects only [15,16] or use BMI as a continuous variable [12]. Failure to include all three categories, normal (BMI < 25 kg m^-2^), overweight and obese, results in an incomplete picture of the misclassification bias associated with self-reported versus measured BMI in studies addressing the prevalence of overweight and obesity.

Evidence suggests that inaccuracies in obesity prevalence estimates based on self-reported height and weight may be compounded in recent years by the increased influence of social desirability on self-reports [3]. Connor Gorber et al. [3] reports that the influence of social desirability on self-reports has the potential to change over time as social and cultural norms about weight and obesity change. This is particularly relevant in the Irish context where the underlying prevalence of overweight and obesity is increasing steadily[17]. While misclassification bias associated with self-reported versus measured BMI has been well investigated, comparatively, we have found only one study[9] that has focused on the changes in self-reported biases across time. This recent Canadian-United States comparison study found that across time the differential between self-reported and measured obesity, derived from height and weight, increased in Canada but remained stable in the US. An older 2009 study from the United States[6], with the potential to discuss temporal trends, pooled their data from 2001-2006. The most recent systematic review [3], due to lack of complete information, was unable to pool results to investigate reporting bias across time.

A recent British study [7] reported a marked decline in sensitivity with respect to individuals' capacity to detect their own overweight and a concurrent improvement in specificity, with fewer people of normal or low weight believing themselves to be overweight. This was a self-classification study as to whether or not individuals saw themselves as overweight/obese. It is suggested that this change in perception of overweight and obesity has important implications for health promotion strategies to combat obesity. The study was based however on a comparison of perceived relative weight versus self-report based BMI categories, the latter used as a gold standard true classification rather than a proxy measurement. We advance this study using BMI calculated from self-reported height and weight and clinically measured BMI, in subsamples from three national lifestyle surveys in Ireland, spanning a period of 10 years. The aim of our study was to evaluate the misclassification biases of all three BMI categories across time using measures of sensitivity and specificity based on self-report based BMI (proxy) compared against clinically measured BMI (gold standard).

## Methods

### Participants and Data

The data used in this analysis were obtained from physical examination subsamples from three national health and lifestyle surveys in Ireland. The Survey of Lifestyle Attitudes and Nutrition (SLÁN) was first conducted in 1998 [18] (n = 6539), and repeated in 2002 [19] (n = 5992) and 2007 [17] (n = 10364). The methods have been described previously[18-20]. Briefly, the 1998 and 2002 surveys consisted of a multi-staged random sample using district electoral divisions (DEDs) across the 26 counties of the Republic of Ireland as the primary sampling units. A self-administered postal questionnaire was distributed to adults aged 18 years and over, and response rates of 62% and 53% were recorded. Although a response rate of 53%-62% is relatively low, it is acceptable by standards of a postal questionnaire and there is evidence that the data are representative. For instance, estimates of macro-nutrient intake derived from the SLÁN 1998 Food Frequency Questionnaire compare very well with the North/South Ireland Food Consumption Survey, a methodologically rigorous survey of 1379 adults employing a 7-day food diary method [21]. There was also remarkable between-survey consistency in some variables between 1998 and 2002, so that data could be pooled for analysis purposes [22-24]. Accordingly we are reasonably confident that the datasets give a reasonable profile of the Irish population at each time period.

The self-reported and measured height and weight data for SLÁN 1998 and SLÁN 2002 were obtained from an out of sample, 10% equivalent of the main postal survey, (SLÁN 1998 n = 586; SLÁN 2002 n = 411). Two DEDS, one rural and one urban, were randomly selected from each Health Board District in Ireland. Letters of invitation, which made reference to a physical examination as a component of the study, were forwarded to potential participants and a nurse followed with a phone call to schedule an appointment within 7 days. Following completion of the SLÁN questionnaire, which included self-reporting their height and weight, height and weight were measured by nurses given specific training and based on documented standard procedures.

SLÁN 2007 consisted of a probabilistic sample in three stages - geographic area, household and 'next birthday' participant selection within households. The sample frame was the Geodirectory, a listing of all residential addresses in Ireland compiled by the postal service. Face-to-face interviews were conducted with adults aged 18 years and over interviewed at home addresses (response rate of 62%). All participants were asked to self-report their weight without clothes and their height without shoes. Examination data were obtained on an approximate 20% subsample, n = 2174. Respondents provided self-reported data at interview before they were asked to agree to have their height and weight measured. Weight and height were measured in light clothing without shoes. Weight was measured to the nearest 0.1 kg using electronic platform scales. Height was measured to the nearest 0.1 cm using height measurement rods. Data were missing for some measured weight, measured height, self-reported weight and self-reported height variables in each of the SLÁN surveys leading to 208 (M 66; F 142), 331 (M 147 F 184) and 2037 (M 909 F 1128) complete cases for comparison respectively in 1998, 2002 and 2007.

### Statistical Methods

BMI values were calculated from self-reported and clinically measured heights and weights. For the purposes of this study, self-report BMI refers to BMI calculated from self-reported heights and weights, as is standard practice in the literature [4,5,7,9,13,16,25-27]. To determine if misclassification differed statistically significantly across characteristics such as gender, education etc., significant differences in the mean misclassification bias were evaluated using a t-test, or one-way ANOVA where appropriate, for the SLÁN 2007 data. Respondents were classified into the BMI categories normal (< 25 kg m^-2^), overweight (25- < 30 kg m^-2^) and obese (≥ 30 kg m^-2^) using both self-reported and clinically measured BMI values. Patterns of reporting bias were displayed by cross-classifying the clinically determined categories with the self-report categories for each SLÁN subsample, and repeated for males and females separately. All results are stratified by gender due to significant differences (*p *< 0.001) in the distribution of under- and overreporting. The sensitivity (probability of a true positive) and specificity (probability of a true negative) of each BMI category were calculated and expressed as percentages. In order to ensure that the endpoints of the corresponding 95% confidence intervals (CI) for these parameters did not exceed 100% Wilson's score interval [28] for a binomial proportion was used. Interaction plots of sensitivity and specificity over time by BMI category were used to show changes in the misclassification patterns across the three SLÁN surveys. Plots of self-reported BMI against clinically measured BMI were used to identify patterns in the reporting biases associated with these variables. A chi-squared test of independence between SLÁN survey and the distribution of misclassifications was conducted. Finally, a chi-squared test for trend in proportions was applied to the proportion under-reporting across the three SLÁN surveys. All statistical analysis was performed using R 2.10.1 [29]. SLÁN 1998 and SLÁN 2002 received ethics approval from the Faculty of Public Health Medicine, Royal College of Physicians of Ireland and SLÁN 2007 was approved by the Research Ethics Committee of the Royal College of Surgeons of Ireland. The data from all three SLÁN surveys are now publicly available.

## Results

### BMI measurement bias

T-tests and one-way ANOVAs were used on the SLÁN 2007 data to investigate if BMI measurement bias differed significantly by the sociodemographic characteristics gender, social class, self-rated health, education, smoking, age group and BMI group (measured). The results indicate that the mean BMI measurement bias varied by education, age group and BMI group (*p *< 0.05). Analysis of the residuals showed that the mean measurement bias was highest in those with some or completed primary education and lowest in those with some or completed tertiary education. As education increases, mean measurement bias decreases. A one-way ANOVA of mean BMI measurement bias and age showed that those aged 18-29 years had the lowest mean measurement bias and those aged 65-80 years had the highest. Measurement bias increased with age to 80 years. There were insufficient subjects in the over 80 years age group to permit comment on this group. Finally the mean measurement bias was lowest for those in the normal category of measured BMI and highest for those in the obese category. As measured BMI category increases, mean BMI measurement also increases.

### BMI misclassifications

**Figure 1:**
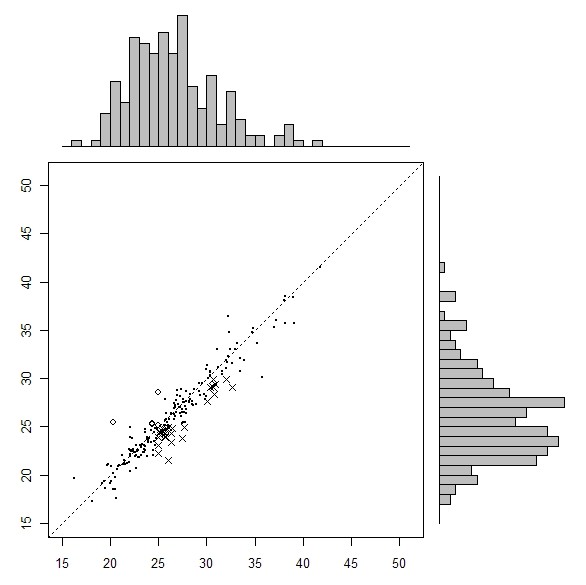
**Measured (x axis) versus self-reported (y-axis) BMI, SLÁN 1998**. Solid dot represents true classifications. x represents BMI misclassifications (underreported), o represents BMI misclassifications (overreported).

**Figure 2:**
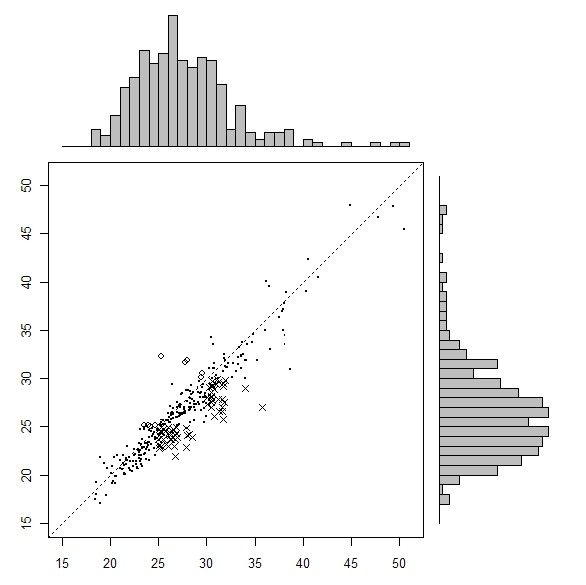
**Measured (x axis) versus self-reported (y-axis) BMI, SLÁN 2002**. Solid dot represents true classifications. x represents BMI misclassifications (underreported), o represents BMI misclassifications (overreported).

**Figure 3:**
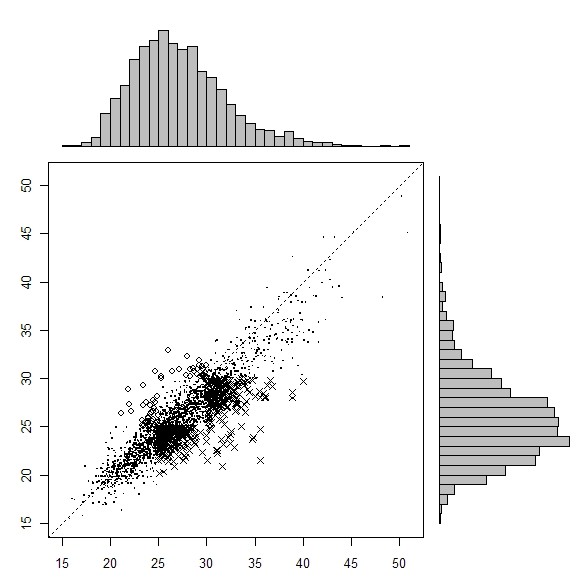
**Measured (x axis) versus self-reported (y-axis) BMI, SLÁN 2007**. Solid dot represents true classifications. x represents BMI misclassifications (underreported), o represents BMI misclassifications (overreported).

The categorical definitions of obesity obtained from self-reported and clinically measured BMIs are compared in Tables 1, 2 and 3 for all three SLÁN surveys. Figures 1, 2 and 3 show a scatter plot with histograms for the three successive surveys with BMI over and under reporters highlighted. Most striking is the greater number of X symbols in all three figures, highlighting the scale of the BMI underestimation problem.

**Table 1:** Cross-tabulations, sensitivity and specificity, of measured and self-reported BMI for all respondents, males and females, in SLÁN 1998

**All Subjects**		BMI Groups (Self-report)			
		**Normal**	**Overweight**	**Obese**	Totals
BMI Groups (Measured)	**Normal**	78 (94)*	5 (6)	2 (2.2)	83 (39.9)
	**Overweight**	20 (24.7)	61 **(75.3)**	9 (8.7)	81 (38.9)
	**Obese**	0 (0)	9 (20.5)	35 **(79.5)**	44 (21.2)
	Totals	98	75	35	208 (100)
	**Specificity**	**84**	**89**	**100**	
**Males**		BMI Groups (Self-report)			
		**Normal**	**Overweight**	**Obese**	Totals
BMI Groups (Measured)	**Normal**	18 **(94.7**)	1 (5.3)	0 (0)	19 (28.8)
	**Overweight**	9 (28.1)	23 **(71.9)**	0 (0)	32 (48.5)
	**Obese**	0 (0)	3 (20)	12 **(80)**	15 (22.7)
	Totals	27	27	12	66 (100)
	**Specificity**	**80.9**	**88.2**	**100**	
**Females**		BMI Groups (Self-report)			
		**Normal**	**Overweight**	**Obese**	Totals
BMI Groups (Measured)	**Normal**	60 **(93.8)**	4 (6.2)	0 (0)	64 (45.1)
	**Overweight**	21 (22.4)	38 **(77.6)**	0 (0)	49 (34.5)
	**Obese**	0 (0)	6 (20.7)	23 **(79.3)**	29 (20.4)
	Totals	71	48	23	142 (100)
	**Specificity**	**85.9**	**89.2**	**100**	

*Sensitivity for normal, overweight and obese categories is represented in parenthesis in bold figures.

**Table 2:** Cross-tabulations, sensitivity and specificity, of measured and self-reported BMI for all respondents, males and females, in SLÁN 2

**All Subjects**		BMI Groups (Self-report)			
		**Normal**	**Overweight**	**Obese**	Totals
BMI Groups (Measured)	**Normal**	102 (96.2)*	4 (3.8)	0 (0)	106 (32)
	**Overweight**	40 (28.8)	94 **(67.6)**	5 (3.6)	139 (42)
	**Obese**	0 (0)	31 (36)	55 **(64)**	86 (26)
	Totals	142	129	60	331 (100)
	**Specificity**	**82.2**	**81.8**	**98**	
Males		BMI Groups (Self-report)			
		**Normal**	**Overweight**	**Obese**	Totals
BMI Groups (Measured)	**Normal**	30 **(100)**	0 (0)	0 (0)	30 (20.4)
	**Overweight**	20 (26.7)	55 **(73.3)**	0 (0)	75 (51)
	**Obese**	0 (0)	17 (40.5)	25 **(59.5)**	42 (28.6)
	Totals	50	72	25	147 (100)
	**Specificity**	**82.9**	**76.4**	**100**	
**Females**		BMI Groups (Self-report)			
		**Normal**	**Overweight**	**Obese**	Totals
BMI Groups (Measured)	**Normal**	72 **(94.7)**	4 (5.3)	0 (0)	76 (41.3)
	**Overweight**	20 (31.2)	39 **(60.9)**	5 (7.8)	64 (34.8)
	**Obese**	0 (0)	14 (31.8)	30 **(68.2)**	44 (23.9)
	Totals	92	57	35	184 (100)
	**Specificity**	**81.5**	**85**	**96.4**	

*Sensitivity for normal, overweight and obese categories is represented in parenthesis in bold figures.

**Table 3:** Cross-tabulations, sensitivity and specificity, of measured and self-reported BMI for all respondents, males and females, in SLÁN 2007

**All Subjects**		BMI Groups (Self-report)			
		**Normal**	**Overweight**	**Obese**	Totals
BMI Groups (Measured)	**Normal**	692 (94.3)*	41 (5.6)	1 (0.1)	734 (36)
	**Overweight**	248 (30.8)	531 **(66)**	26 (3.2)	805 (39.5)
	**Obese**	20 (4)	212 (42.6)	266 **(53.4)**	498 (24.4)
	Totals	960	784	293	2037 (100)
	**Specificity**	**79.4**	**79.5**	**98.2**	
**Males**		BMI Groups (Self-report)			
		**Normal**	**Overweight**	**Obese**	Totals
BMI Groups (Measured)	**Normal**	242 **(92.4)**	20 (7.6)	0 (0)	262 (28.8)
	**Overweight**	101 (24.4)	296 **(71.5)**	17 (4.1)	414 (45.5)
	**Obese**	5 (2.1)	101 (43.3)	127 **(54.5)**	233 (25.6)
	Totals	348	417	144	909 (100)
	**Specificity**	**83.6**	**75.6**	**97.5**	
**Females**		BMI Groups (Self-report)			
		**Normal**	**Overweight**	**Obese**	Totals
BMI Groups (Measured)	**Normal**	450 **(95.3)**	21 (4.4)	1 (0.2)	472 (41.8)
	**Overweight**	147 (37.6)	235 **(60.1)**	9 (2.3)	391 (34.7)
	**Obese**	15 (5.7)	111 (41.9)	139 **(52.5)**	265 (23.5)
	Totals	612	367	149	1128 (100)
	**Specificity**	**75.3**	**82.1**	**98.8**	

*Sensitivity for normal, overweight and obese categories is represented in parenthesis in bold figures.

Across the three successive surveys, misclassification bias was most evident in the obese category in SLÁN 2007 (Table 3). The sensitivity was 53.4%, down from 64% in 2002 and 79.5% in 1998. The majority of misclassifications were in the overweight category. Gender differences were minor. Underestimation of BMI in all three surveys was also evident in the overweight category. In SLÁN 2007 and 2002, BMI underestimation was higher for females than males in the overweight category.

Table 2 shows that in SLÁN 2002 self-reported BMI was underestimated when compared to the 'gold standard' clinical measurement. Misclassifications were greatest in the obese category. 64% of obese respondents (n = 86) correctly self-reported themselves as obese and 36% incorrectly reported themselves as overweight. Higher proportions of females correctly classified themselves as obese; the sensitivity for females in the obese category was 68.2% versus 59.5% for males. In both cases, the proportion of normally weighted respondents reporting themselves as normal weight is high, 100% for males and 94.7% for females.

The clinically measured and self-report BMI classifications of each subject were combined into a single polychotomous variable called 'misclassification' with levels underreported, correctly reported, and over reported (Table 4). A chi-squared test of independence between SLÁN survey and the distribution of misclassifications gave χ^2 ^= 11.676 (df = 4, *p *= 0.02). The percentage of subjects underreporting their height and weight leading to an incorrect BMI classification increases from 14% to 21% to 24% across the three SLÁN surveys. A chi-squared test for trend in proportions was applied to the proportion underreporting across the three surveys and was statistically significant at the 5% level (*p *= 0.002).

**Table 4:** Number and proportion of misclassifications by SLÁN survey

	Misclassification		
	Under reported	Correctly reported	Over reported
**SLÁN 98**	29 (14)	174 (84)	5 (2)
**SLÁN 02**	71 (21)	251 (76)	9 (3)
**SLÁN 07**	480 (24)	1489 (73)	68 (3)

### Sensitivity and Specificity Trends

**Figure 4:**
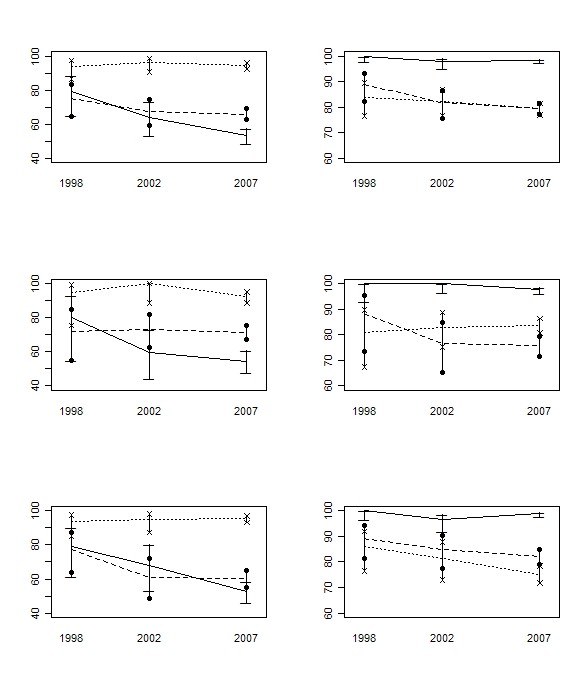
**Plots of sensitivity (left column) and specificity (right column), with 95% CI against SLÁN survey (x-axis): all respondents (1^st ^row), males (2^nd ^row), females (3^rd ^row); normal (^..............^), overweight (--------), obese (^_______^)**.

The sensitivity and specificity for each BMI category across time are plotted, with 95% confidence interval bands, in figure 4 for the three SLÁN surveys. In all three, sensitivity was highest for males and females in the normal category indicating that men and women of normal weight were least likely to have been allocated to the wrong BMI category. Simultaneously the specificity was high, exceeding 80% at all times. Confidence interval bands around these calculations were narrower than for either the overweight or obese categories.

While sensitivity for the overweight category decreased across time, indicating an increase in misclassifications, this stabilised between the latter two surveys for both males and females (figure 4, left column). However, the number of false negatives was greater for females than males in the 2002 and 2007 surveys. The sensitivity for males exceeded 70% while for females it was 61% Specificity decreased in the overweight category across time, indicating an increase in the number of false positives. In all three surveys, specificity was higher for females than males.

Sensitivity decreased significantly in the obese category across time. Gender variability was evident in 2002 but was relatively equal in 2007, 54.5% and 52.5% for males and females respectively. This indicates that almost half of the respondents who are obese based on measured values are not categorised as obese by self-report values. Simultaneously the specificity for the obese category remained high and exceeded 98% in all three surveys. This indicates a very small number of males and females are incorrectly categorised as obese (false positive).

### BMI trends

The estimated prevalences of overweight and obese were notably greater when calculated from measured values rather than self-reported values, indicating a bias and a high probability of misclassification. Self-report BMI values indicate that though an increase was observed in 2002, overall obesity levels in Ireland have decreased in the last 10 years; 16.8%, 18.1% and 14.4% in the three successive surveys. Trends in the clinically measured data show an increase; 21.2%, 26.0% and a minor fluctuation to 24.4% in 2007 (*p *= 0.925). According to self-report data, overweight levels increased from 36.1% to 39% in 2002 and remained stable in 2007. Measured values showed an increase between 1998 and 2002, 38.9% to 42% and a decrease to 39.5% in 2007. These findings were not statistically significant.

## Discussion

### Principal findings

We find that the differential between BMI derived from self-reported height and weight and BMI derived from measured height and weight is increasing across time, a finding supported by Canadian data [9] but at variance with both Swiss [10] and US data [9] in which the differential has remained constant over time. We found declining sensitivity scores for overweight and obese categories, when self-reported BMI was compared to measured BMI across time. The declining sensitivity, accompanied by rising levels of obesity in the population, lends support to the suggestion by Connor Gorber et al. [3] that the influence of social desirability on self-reports is changing, as social and cultural norms about weight and obesity are changing. Using data from three surveys in Ireland, notwithstanding the extremely small sample sizes in the first two surveys, we find that BMI values based on self-reported determinations of height and weight are underestimating the true prevalence of the obesity epidemic. By measured data from SLÁN 2007, 64% of the Irish adult population are overweight or obese.

### Comparison with other studies

In our study, obesity prevalence based on measured values (measured BMI - self-reported BMI) was 4.4%, 7.9% and 10.0% greater for the three surveys respectively, indicating an increased differential across time. The chi-squared test for proportions, applied to the proportion of under reporters across the three successive SLÁN surveys is also statistically significant. Though it is well reported, and confirmed in a recent systematic review [3] that self-reported BMI is most often lower than measured BMI, the finding that this differential is increasing over time is relatively new. This trend was first identified in a 2010 study of Canadian data [9], but the same study found that an increased differential between self-reported and measured BMI across time was not discernable in US data. Our study is on a par with the findings of the Canadian data where the discrepancy for the obesity category was large and has doubled from 4% to 8% in the past decade.

A decline in the sensitivity of the obese category, coupled with rising obesity levels, has important implications for public health professionals working to combat the rising obesity levels. Misclassifications to a lower BMI category will exaggerate the association between obesity and obesity related conditions [30-32]. This is a consequence of non-random misclassification of self-reported BMI [30]. Moderately obese individuals on the overweight/obese threshold are more likely to be categorised as overweight thereby inflating the estimates of risk associated with overweight. This non-random misclassification will also inflate the risk estimates associated with obesity, as the effective threshold for the latter will be above 30 kg m^-2 ^[30-32].

We found the burden of misclassification could not be attributed to either sex, contrary to a previous British study that reported a sensitivity of 59.1% for obese males compared to 73% for obese females [13]. Across our three surveys, a trend related to gender bias was not evident.

The recent report on misclassification bias by Johnson et al. [7] using sensitivity and specificity as a measure, found a marked decline in sensitivity with respect to individuals' detection of their own overweight. Though different to the current study, in that it was a self-classification study as to whether or not individuals saw themselves as overweight/obese, a key limitation of the study was the inability to distinguish between overweight and obese categories of BMI. Furthermore, the comparison of perceived relative weight ('underweight', 'about the right weight', 'somewhat overweight', 'very overweight', and including 'obese' in 2007 only) to self-report BMI categories ('underweight', 'normal weight', 'overweight', 'obese') the latter used as a gold standard true classification rather than a proxy measurement, was a further restraint. We have advanced the Johnson et al. [7] study by using a gold standard based on actual measurement of BMI and a self-report measure of BMI from self-reported height and weight, for a direct comparison between BMI categories. A particular strength of the present study is our ability to cross classify all three BMI categories and avoid dichotomising BMI. This has resulted in the important finding that there has not been a continuing decrease in sensitivity in the overweight category, a pertinent difference in the two studies.

The sensitivity values consistently exceed 94% in the normal category indicating that men and women of normal BMI are least likely to incorrectly self-report their height and weight. That this finding is consistent over time is important for future studies targeting self-reported misclassification bias, and those considering a correction factor. This finding has previously been observed in other studies. The systematic review of women's height and weight reported that in 19 of 20 studies, heavier women reported their weight less accurately [33]. Other studies have also reported that underreporting of weight increases with increasing measured weight [3,13,14].

### Implications for research and practice

The findings of this study support other published work questioning the accuracy of self-reported BMI in research studies used to determine overweight and obesity levels in general populations. However, the results also suggest a finding that was not altogether expected, that where accuracy is of prime concern, i.e. the obese category, biases are increasing. Given that Canadian data and now Irish data show an increase in BMI underestimation across time in the obese category, the size of this underestimation should be monitored and taken into consideration when planning public health policies related to overweight and obesity.

It is unclear why the very group that are much in need of intervention do not see themselves as so. Possibilities cited in previous literature [7,34] may also hold true in our study. It may be that this group are aware of their body weight but do not want to be labelled as 'obese' given the negative connotations associated with obesity and the media portrayal of 'obesity', 'obesogenic environments' and 'morbidly obese' people. At a national level the Irish government established a national task force on obesity, which produced a report in 2005 [35] with over ninety recommendations for cross-sectoral action, and this received much public attention. It is also possible that the group are in denial of their unhealthy weight. The third and most distinct possibility does not concern acceptability but rather a genuine shift in the normative definitions of overweight and obesity, driven by social change. International data support this theory [7,34]. As our 2007 measured data show, one in four Irish adults are obese and a large majority, 64% of our study population, are either overweight or obese. These findings, coupled with the declining sensitivity in the obese category over time, suggest that as our sense of what is normal, overweight and obese is changing around us, Irish people are finding it increasingly difficult to make accurate estimates of their height and weight, leading to an increase in underreporting of BMI across time. This trend has also been observed in Canada.

While it is clear that in all countries people find it difficult to make accurate estimates of their height and weight, why we should see an increase in the differential between self-reported height and weight and measured height and weight across time in Ireland and Canada, and a consistent level of underreporting in both the US and Switzerland, is uncertain. With respect to the acceptability of obesity in society, it could be argued that being obese, and certainly being overweight has become more acceptable, and since social norms tend to adjust to average values, we should expect to see a decline in the self-report misclassification bias. However, data from the four countries do not support this. Further studies from other countries are needed to give a cogent explanation of these findings.

The implications for practice are clear; BMI determined from self-reported height and weight is no longer suitable to monitor obesity trends in populations. Project investigators should endeavour to gain accurate measures of BMI using clinical measurement to continue to monitor obesity trends.

### Possible weaknesses of the study

Data collection methods were not identical in the three surveys. SLÁN 1998 and SLÁN 2002 measurement samples represent out of sample groups while the SLÁN 2007 measurement sample is a subsample of the main survey. However data from the main SLÁN surveys are reasonably comparable to the contemporaneous census data. For present purposes, we believe the different sampling methods did not influence the overall premise of the results.

Participants in SLÁN 1998 and SLÁN 2002 were aware that they would undergo a physical examination, after completion of the self-report questionnaire. They may have been aware therefore that their height and weight would be measured after completing the questionnaire. Participants in SLÁN 2007 were not aware that they would undergo a physical examination until the self-report questionnaire was completed. While the most up to date systematic review [3] discusses the order of data collection, it does not discuss knowledge of an impending physical examination in cases where the self-report measurement came first. We can postulate as to the possible influence of this knowledge, or indeed if there is any influence at all. It is possible that having knowledge of the impending physical examination would lead to more accurate self-reports from the 1998 and 2002 SLÁN respondents when compared to the 2007 respondents. However, the order of measurement was the same and the time elapsed for measurement was the same and the upward misclassification bias trend was already evidenced between 1998 and 2002. Therefore we do not believe that knowledge of the impending physical measurement accounts for the trends observed in our study.

In SLÁN 1998, SLÁN 2002 and SLÁN 2007, the response rates were 62%, 53% and 62% respectively. Although these response rates are relatively low, and declining response rates are a feature of national health and lifestyle surveys in industrialised countries, we have provided evidence in the methods section that the data are representative of the Irish population in general. We wish to be clear that a distinction be made between representativeness generally of the SLÁN datasets and what we assert in the within-individual sub-group comparisons. The subsets for whom examination data were collected may not themselves necessarily be representative of the general population, though we have no reason to believe they are not, but they do show how within-individual self-estimates have shifted over time. However, the sample sizes are small in the first two SLÁN surveys and caution is needed in drawing inferences on trends in misclassification across the three surveys.

Information on the differences between responders and non-responders for the SLÁN surveys is not available for evaluating the potential bias due to non-response. It is possible therefore that individuals who significantly underreport their weight may not have consented to measurement.

Measurement bias is a consideration in this study but weight and height data in SLÁN were measured by trained personnel using standardised equipment following a standard protocol thereby minimising any potential measurement biases.

## Conclusions

BMI values based on self-reported determinations of height and weight in population samples are underestimating the true prevalence of the obesity epidemic and this underestimation is increasing with time. Self-report based BMI is not a reliable estimate of obesity prevalence and is an unsuitable measurement method going forward. The decreased sensitivity and consistently high specificity scores in the obese category across time, highlights the limitation of self-report based BMI classifications and the need for simple, readily comprehensible indicators of obesity.

## Competing interests

The authors declare that they have no competing interests.

## Authors' contributions

FS worked as a senior researcher on the 1998 and 2002 SLÁN data, worked on the statistical analysis with KH and drafted the paper. KH was the statistical consultant for this paper and conducted the statistical analyses of the data with FS. IJP was a PI on SLÁN 2007 and was a contributor to the study design, data analysis and interpretation of the main report. He made revisions to the paper. JL is an author on the main SLÁN 2007 report and had a major role in the data analysis and interpretation therein. She made revisions to the paper. JH is an author on the main SLÁN 2007 report and had a major role in the data analysis and interpretation. She made a substantial contribution to the design of the physical examination data collection methods in SLÁN 2007. She made revisions to the paper. CCK was a principal investigator on SLÁN 1998 and 2002. She made revisions to the paper. HMG was lead principal investigator on SLÁN 2007, made a substantial contribution to the design of the physical examination data collection methods and was a contributor to the main report. She made revisions to the paper. All authors approved the final version of the paper for publication.

## Pre-publication history

The pre-publication history for this paper can be accessed here:

http://www.biomedcentral.com/1471-2458/10/560/prepub
